# Small‐diffusible aggregates, plaques, tangles, and dynamic equilibria: Untangling Alzheimer's disease

**DOI:** 10.1002/alz.70462

**Published:** 2025-07-03

**Authors:** Emre Fertan, Georg Meisl, David Klenerman

**Affiliations:** ^1^ Yusuf Hamied Department of Chemistry University of Cambridge Cambridge UK; ^2^ UK Dementia Research Institute at University of Cambridge Cambridge UK; ^3^ Present address: Department of Clinical Neurosciences, University of Cambridge, Cambridge, UK

**Keywords:** Alzheimer's disease, beta‐amyloid, caspase, microglia, oligomer, plaque, post‐translational modification, small‐diffusible aggregate, tangle, tau, truncation

## Abstract

**INTRODUCTION:**

Beta‐amyloid plaques and hyperphosphorylated tau tangles are the neuropathological hallmarks of Alzheimer's disease; however, their relevance in the pathophysiology is not fully understood. It has been suggested that these larger and insoluble aggregates may not be the most toxic forms of beta‐amyloid and tau in Alzheimer's disease, and the disease progression may actually be promoted by the small‐diffusible aggregates.

**METHODS AND RESULTS:**

We combine the recent findings from our group and other key research to put forward the hypotheses that the formation of the small‐diffusible aggregates of beta‐amyloid and tau and their larger insoluble counterparts is not a linear process.

**DISCUSSION:**

While the small‐diffusible aggregate formation of beta‐amyloid and tau is a passive process, regulated by thermodynamic equilibria, the formation of large‐insoluble aggregates is an active process, regulated by microglia and neurons, which to an extent is a protective mechanism against the toxicity of the smaller aggregates.

**Highlights:**

- Plaques and tangles may be made by active processes in Alzheimer's disease.
- The small‐soluble aggregates may be the more toxic species in Alzheimer's disease.
- Pathology may be caused by the imbalance of production and clearance of aggregates.
- Plaques and tangle formation may be attempts to restore the homeostatic equilibrium.

## 1 INTRODUCTION

Alzheimer's disease (AD) is the globally leading cause of dementia with neuropathological symptoms of synaptic loss, gliosis, and cerebral atrophy, which collectively lead to cognitive and behavioral dysfunction.^1^ The total[Fig alz70462-fig-0001] number of dementia patients worldwide is currently estimated as 55 million,^2^ and according to the United Kingdom Alzheimer's Society, there were over 980,000 people with dementia in the United Kingdom in 2024 (50%–75% of the cases being AD).^2^, ^3^ AD is a debilitating condition that affects the individual, their families, and society as a whole, making it essential to gain knowledge about the disease and develop effective treatments.

Even though its presence was documented by ancient Greeks and Romans,^4^ AD was first systematically studied and described by German neuroanatomist and psychiatrist Alois Alzheimer following observations of severe neuronal loss and seed‐like formations caused by the deposition of a “special substance.”^5^ Later in the 1980s, Konrad Beyreuther and colleagues identified the “special substance” as beta‐amyloid (Aβ)^6^ and linked it to a larger precursor protein located on the 21st chromosome,^7^ which is today known as the amyloid precursor protein (APP). These observations eventually lead to the amyloid cascade hypothesis, suggesting the abnormal accumulation of Aβ as the initial cause of pathogenesis in AD^8^, ^9^ and the initiator of the co‐pathologies including inflammation,^10^ oxidative damage,^11^ and hyperphosphorylated tau aggregate accumulation,^12^ which is the second neuropathological hallmark of AD.

**FIGURE 1 alz70462-fig-0001:**
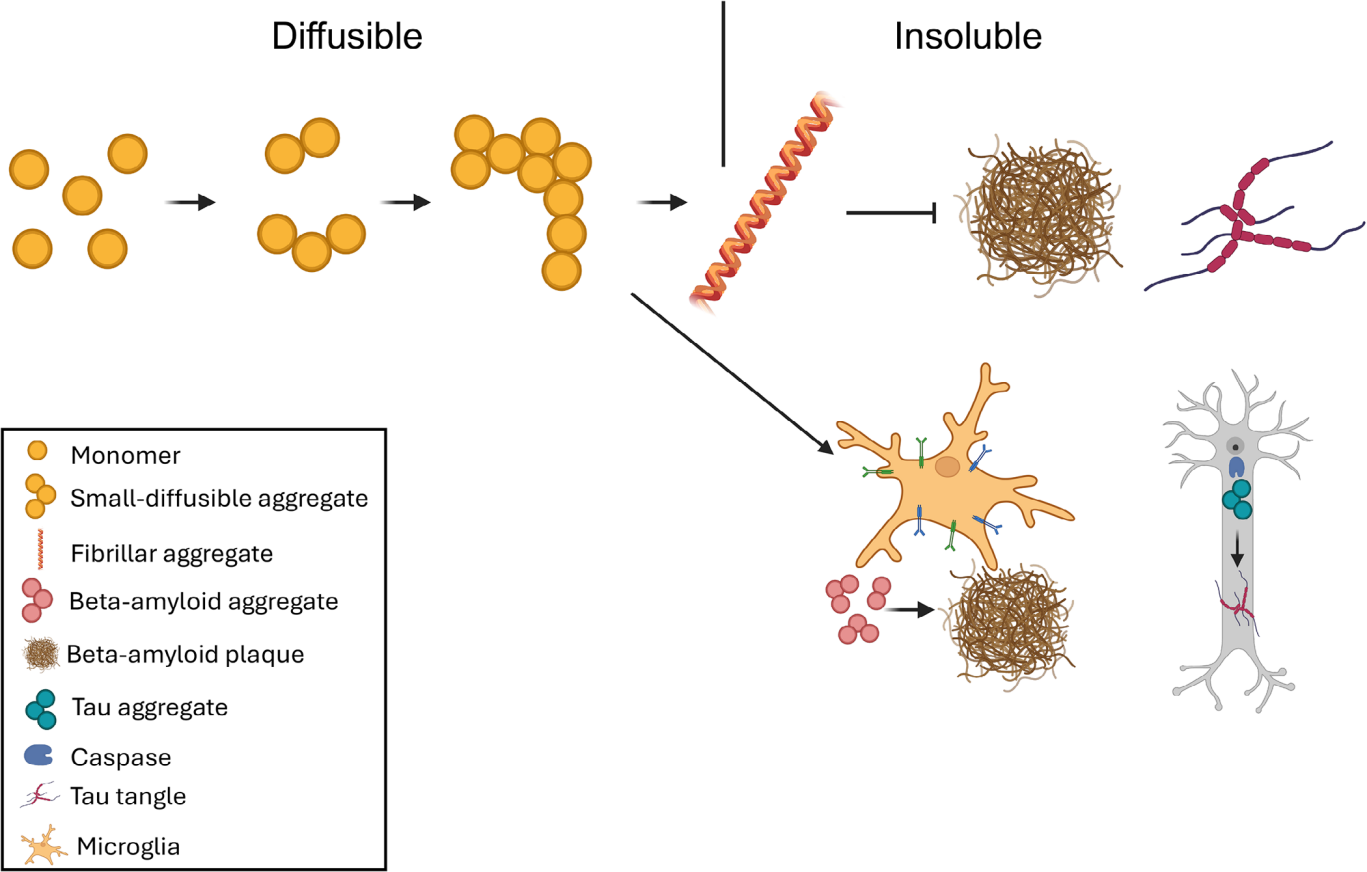
Formation of beta‐amyloid and tau aggregates in Alzheimer's disease. Initial aggregation follows biophysical models, initiated by the monomer concentration exceeding its solubility threshold, forming dimer, trimers, and eventually oligomers (soluble species) as a continuum. According to our hypothesis, the formation of larger beta‐amyloid plaques and tau tangles made of insoluble fibrils is not a continuation of this passive aggregation process, but active mechanisms regulated by microglia and neurons. (Created in BioRender, Agreement # TV28AAT2EW).

Although a substantial amount of research has been done to study the plaques and tangles, and the atomic structure of the fibrils making up their core components have been determined using cryogenic electron microscopy (CryoEM) in elegant studies identifying “disease‐specific folds,”^13^, ^14^ their role and relevance in the pathogenesis of AD has not been established. The strongest arguments against the amyloid cascade hypothesis are the presence of Aβ plaques in the brains of cognitively intact individuals^15^, ^16^ and failure of therapeutics targeting the plaques^17^ (with the exception of donanemab, discussed further below), together suggesting that Aβ may not be causing the AD pathology. Meanwhile, recent studies have indicated that the large and insoluble plaques may not be the primary toxic forms of Aβ aggregates, but the smaller and diffusible aggregates (sometimes called “oligomers”) may be the more toxic species^18^ and targeting these smaller aggregates can provide more beneficial therapeutic outcomes.^19^ While the plaques are well above the diffraction‐limit of light and thus relatively easier to observe with traditional light‐microscopy techniques, the diffusible aggregates are on average around 100 nm in length and require special single‐molecule methods to be studied and characterized.^19^, ^20^, ^21^ A key question that needs to be answered to further understand AD pathogenesis and develop yet more effective treatments is the mechanism of formation of both the small‐diffusible aggregates and the larger neuropathological hallmarks. They may sit on a continuum from monomers to small‐diffusible aggregates to fibrils, a common assumption in the field, or separate mechanisms may give rise to diffusible aggregates and the insoluble plaques and tangles (Figure 1). In this perspective, we discuss this question, based on recent findings from our laboratory and others, and the consequences of active fibril formation.

### 1.1 Small‐diffusible aggregates and plaques

Formation of the small‐diffusible aggregates is explained by biophysical models of aggregation: when a monomeric protein or peptide is present at concentrations above its solubility threshold, thermodynamic forces drive the system to aggregate.^22^ Often, there may be a significant barrier to the formation of the first few aggregates, the nucleation step, which can keep a monomeric system from aggregating. Once an aggregate has formed, it can grow by addition of further monomeric protein from solution and a range of different aggregate sizes and potentially also of different morphologies can form in this way. Moreover, once the first aggregates are present, they can trigger the formation of more aggregates, for example by fragmentation; the breaking of existing aggregates, or secondary nucleation, which is the formation of new aggregates by the surface of existing aggregates functioning as a template for aggregation. First discovered in the aggregation of sickle hemoglobin,^23^ and further demonstrated in more recent studies, the intrinsic ability of aggregates to self‐replicate seems to be a common property across many proteins.^24^ This aggregate formation is a purely passive process that takes place even in a test tube, although in living systems the rate of aggregate formation is likely attenuated by the protein control machinery.

By contrast, the formation of plaques, which consist of many individual Aβ  fibrils along with various other proteins,^25^ may be an active process regulated by microglia. Simple physical principles would support this concept since the extracellular concentration of Aβ42 is in the range of 1–10 nM,^26^ which is below its critical aggregation concentration in the brain (90 nM),^27^ so that spontaneous fibril formation will not occur. Using mouse models of AD, Bart De Strooper and colleagues have recently shown that microglial depletion reduces Aβ plaque formation, while the amount of diffusible aggregates remain unchanged.^28^ Similarly, Greg Lemke and colleagues have shown that expression of the TAM receptor tyrosine kinases Axl and Mer is elevated in mouse models of AD, and their downregulation results in the reduction of dense‐core plaques.^29^ Collectively, the passive formation of small‐diffusible aggregates and the microglia‐driven formation of plaques suggest that although these are not completely independent events, plaques are not formed by the passive continued growth of diffusible aggregates. Combined with the studies showing higher toxicity for small‐diffusible aggregates, active plaque formation by microglia may be an attempt for a protective mechanism, by packing the diffusible aggregates into larger, less bioactive species,^30^ to restore the dynamic equilibrium between the formation and clearance of small‐diffusible Aβ aggregates during early stages of AD. If this attempt is successful, it explains the presence of Aβ plaques in cognitively intact individuals, as well as the long temporal gap between plaque formation and cognitive deficits^31^: disease progression was either halted by restoring the equilibrium in these individuals, or delayed until the saturation of plaques, which would lead to further accumulation of the more toxic small aggregates and eventually cognitive loss if these individuals survived for longer. On the other hand, if the aggregate production continues to exceed clearance, AD continues to progress leading to secondary disease mechanisms, particularly tau aggregation, along with high‐levels of plaque load, accompanying cognitive decline.

### 1.2 Tau aggregates in AD

The second neuropathological hallmark of AD, namely the neurofibrillary tau tangles raise the same question: are the aggregation mechanisms of post‐translationally modified tau, in terms of the formation of small‐diffusible multi‐phosphorylated aggregates and large‐insoluble tangles a continuum or regulated by different mechanisms? We speculate that the high concentration of other proteins that can interact with tau in the cytosol would likely make fibril formation energetically unfavorable requiring the active formation of truncated tau through caspase activation, to lower the barrier of protective fibril formation. Thus, while the formation of diffusible tau aggregates is expected to occur based on the fundamental biophysics of the aggregation process, the larger and insoluble tau tangles may be generated by active processes. Using mouse models of tau pathology and in vivo multiphoton imaging, Bradley Hyman, Tara Spires‐Jones, and colleagues have demonstrated that caspase activation (specifically caspase‐3 and ‐6) indeed precedes tangle formation in living neurons.^32^ Importantly, tangle formation almost always followed caspase activation in these neurons within hours, with a significantly higher occurrence rate compared to neurons without caspase activity, suggesting an active role of caspase activity and tau truncation in tangle formation. Remarkably, caspase activity was diminished following the formation of tangles. Moreover, both in mouse models and *post mortem* AD brain samples, neurons containing tangles were shown to survive^33^ and remain integrated in the neuronal circuits maintaining their function,^34^ while the diffusible‐tau aggregates were shown to be the toxic species.^33^ Indeed, no significant changes were found in the transcriptome profile of tangle‐bearing neurons for genes associated with neuronal death.^35^ Collectively, these results indicate that neurofibrillary tangle formation is an active process, correlating with protease activity and tau truncation, and it is not directly associated with neuronal loss. However, the “chicken‐and‐egg relationship” between surviving the caspase activation and tangle formation needs to be further investigated to determine causality. Since soluble tau aggregate accumulation in the soma can lead to caspase activation promoting cell death^36^ and the formation of tangles suppresses the caspase activity,^32^ the existence of a threshold for soluble tau aggregate levels can be speculated, above which tau becomes the primary target of the activated caspases leading to truncation and tangle formation, resulting in a double‐benefit for the neuron, both reducing the toxic soluble tau levels and silencing the caspase activation, promoting survival. This also explains the high spatial and temporal correlation of tangle formation and neurological deficits, as tangle formation would be a rare event in neurons with caspase activation, which in most cases would lead to cell death.

There is also growing evidence that the most toxic tau species are not the insoluble fibrils, which are capable of seeding. Recently developed mouse models of tauopathy has shown that mutations leading to the accumulation of non‐fibrillar tau causes synaptic loss and behavioral abnormalities, suggesting that these diffusible species are the source of toxicity.^37^ Moreover, electrophysiological recordings from the hippocampal CA1 neurons of the rTg4510 mouse model of tau pathology revealed that deficits in complex‐spike firing precedes tangle formation and primarily promoted by soluble high‐molecular weight tau species, through the reduction in CaV2.3 calcium channels, which could be replicated in wild‐type mice with human AD‐brain derived soluble high‐molecular weight tau aggregates,^38^ providing further evidence for the role of small tau aggregates rather than the tangles in neuronal pathology in AD. Interestingly, when correlated with the age of death in AD patients, insoluble tau showed a positive correlation, with higher levels in patients who survived longer, yet soluble tau showed no correlation and S396 phosphorylated tau was correlated negatively,^39^ further showing the formation of the small‐diffusible and larger tau aggregates is not a continuum and regulated by distinct mechanisms. Collectively, these findings are challenging the relatively older hypotheses of AD, which suggest tangle pathology closely correlates with cortical atrophy and cognitive decline.^40^ Indeed, “ghost tangles,” which are believed to be initially formed intracellularly yet found in the extracellular space due to the death of the neuron, only constitute about 0.7% of all tangles in the *post mortem* human brain tissue,^41^ showing that majority of the neurons with tangles are intact during end‐stage AD.

### 1.3 The multi‐stage dynamic equilibria hypothesis

These findings suggest that AD is a multi‐stage disease of breaking and trying to re‐establish the dynamic equilibrium of formation and clearance of toxic aggregates. Indeed, it has recently been proposed that the failure of age‐related damage clearance mechanisms underly the genetic architecture of AD.^42^ We have shown that a chronic inflammation challenge with tumor necrosis factor alpha can tilt this equilibrium and lead to the production of small‐diffusible Aβ aggregates in wild‐type iPSC‐derived neurons. Importantly, these aggregates are cleared to the media without the initiation of tau pathology,^43^ suggesting a physiological role for Aβ within the central nervous system, working as a damage response element following inflammatory challenges.^10^ However, if the dynamic equilibrium is tilted further and eventually broken, as we have demonstrated with neurons carrying familial AD‐related mutations,^43^ the diffusible Aβ aggregates start to grow larger. This may then cause microglia activation initially working as a protective mechanism either by clearing the diffusible aggregates or packing them into insoluble dense‐core plaques.^28^ As such, plaque formation is not a natural continuum caused by the growth of the diffusible‐aggregates, but an active attempt of restoring the equilibrium. At this point, it should we clarified that we are not hypothesizing that the Aβ plaques are completely benign and protective against diffusible Aβ toxicity. Indeed, it has been shown that the plaques do cause an inflammatory response in microglia in an age‐, sex‐, and genotype‐related manner in mouse models of AD.^44^ Nevertheless, they are less bioactive and thus relatively less toxic than the small‐diffusible aggregates, since a large number of small aggregates have formed one much larger single aggregate that cannot diffuse. Therefore, actively clustering the diffusible aggregates into plaques in a brain where the homeostatic equilibrium of production and clearance has been broken,^42^ is a secondary safety mechanism and an attempt to restore the homeostasis. This explains the observations of cognitively intact individuals with Aβ plaque pathology,^15^ as the reduction of small‐diffusible aggregate concentration by the formation of plaques was successful at restoring the equilibrium and stopped (or delayed, in which case the individual may die before showing cognitive loss in the presence of cerebral Aβ plaques) the co‐pathologies such as small‐diffusible tau aggregate accumulation. However, if this mechanism becomes overwhelmed and the balance between diffusible aggregate production, plaque formation, and clearance is broken, further accumulation of the diffusible aggregates can give rise to tau pathology through various mechanisms including oxidative damage, inflammation, and changes in gene expression. Following the same idea, the hyperphosphorylated tau aggregates, which are synapto‐ and neuro‐toxic, are first cleared and then packed into neurofibrillary tangles to be passivated by neurons through caspase activation. Once again if the balance between production and removal is broken, synaptic and neuronal toxicity is initiated, leading to cognitive and behavioral dysfunction associated with AD. Indeed, it has been shown that the lack of synaptic tau oligomers is a key difference between the individuals with plaque and tangle pathology who do and do not show cognitive deficits,^45^ as the synaptic soluble tau aggregates were associated with microglia and astrocyte mediated synaptic pruning in individuals with cognitive impairment.^46^

### 1.4 Conclusion, limitations, and future directions

**FIGURE 2:**
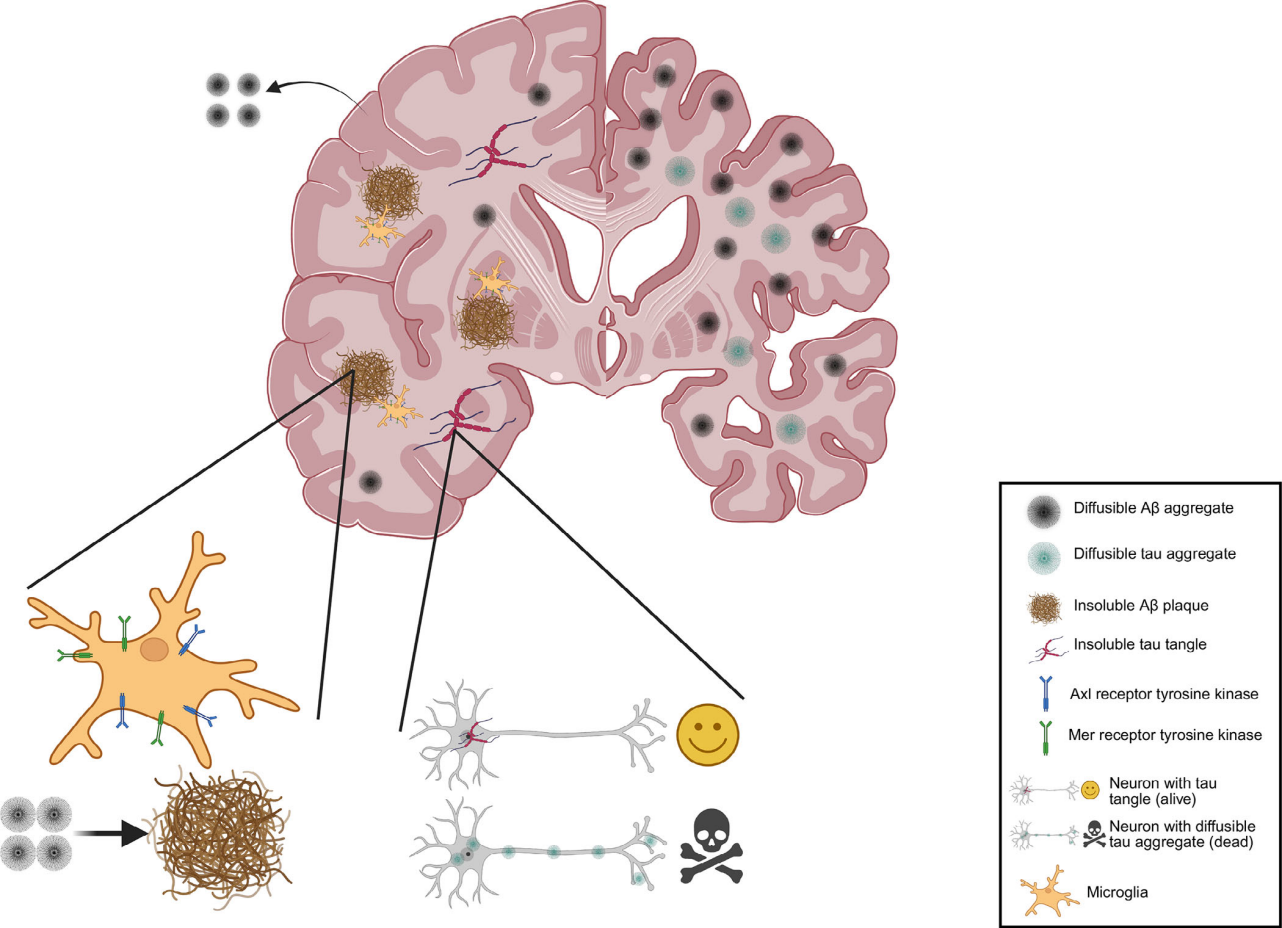
Schematic representation of the multi‐stage equilibria hypothesis. Once the aggregation of beta‐amyloid and tau starts, the small‐diffusible aggregates which are highly bioactive and thus toxic are actively packed into larger insoluble plaques and tangles, which are less toxic, in order to re‐establish the dynamic equilibrium of aggregate production and clearance. If this is not successful and levels of small‐diffusible aggregates continue to increase, this promotes Alzheimer's disease pathology. Figure is not‐to‐scale. (Created in BioRender, Agreement # KP281ATETA).

Collectively, the findings we describe and discuss here challenge some of the long‐standing ideas of AD and the amyloid cascade hypothesis and suggest that, while the initial hypothesis for the role of Aβ in the pathogenesis of AD is indeed correct, our understanding of the small‐diffusible aggregates and their relationship with the large insoluble plaques and tangles needs to evolve (Figure 2). The multi‐stage process of breaking of dynamic equilibria and initiation of secondary toxicity‐reduction mechanisms explain the slow progression of AD and suggest that clearing the diffusible Aβ^19^ and tau^47^ aggregates could be a viable strategy for halting the progression of AD, by re‐establishing the balance between production and removal. One of the predictions of the multi‐stage hypothesis with homeostatic equilibria is the presence of small‐diffusible aggregates in people without AD pathophysiology; AD is not promoted by the production of aggregates, but by the imbalance between the rate of their production and removal. Since the formation and removal of the aggregates is in a dynamic equilibrium at any given time (i.e., at a time of blood or cerebrospinal fluid sample collection, or during the time of death, for *post mortem* tissue sampling), small‐diffusible Aβ and tau aggregates exist in non‐demented individuals; however, the balance between their production and clearance is shifted in AD, favoring the formation of more and/or larger and/or more post‐translationally modified aggregates, which promote the co‐pathologies. As such, protein aggregates are not only features of neurodegenerative conditions, with aggregation happening solely in disease. Small‐diffusible aggregates can indeed be observed both in the nervous system and blood, when the methodologies with sufficient sensitivity and specificity are employed, as we have shown on different occasions for both Aβ and tau.^19^, ^21^, ^48^, ^49^

We are well aware that multiple aspects of the hypothesis we put forward need to be further tested and supported with experimental data, even though there already exists a growing body of literature showing the toxicity of these oligomeric aggregates. Small‐diffusible Aβ aggregates have been shown to cause toxicity intracellularly through interaction with specific DNA sequences and epigenetic regulation of AD‐related genes in mouse and cellular model, as well as the human brain^11^, ^50^ and synaptic dysfunction by regulating AMPA receptors^51^ and translocating tau to the synapse in cultured murine cortical neurons.^52^ In parallel, extracellular diffusible aggregates can interact with numerous receptors including the glial toll‐like receptor 4, promoting inflammation, long‐term potentiation deficits,^53^ alpha seven nicotinic receptors to alter cholinergic neuron activity,^54^ as well as causing glycogen synthase kinase‐3 (GSK‐3β) signaling deficits and promote tau phosphorylation in human neural stem cells.^55^ Meanwhile, small‐diffusible tau aggregates are known to promote inflammation, oxidative damage, and neuronal senescence and loss.^56^ These mechanisms of toxicity and how they are caused by small‐diffusible aggregates needs to be further explored. Indeed, insoluble Aβ and tau aggregates have also shown to induce toxicity, indicating that they are not entirely benign. Microglia activation and initiation of a disease phenotype has been demonstrated in a spatial proximity dependent manner to the Aβ plaques, using spatial transcriptomics,^57^ although this may at least partly be due to the presence of oligomeric aggregates around the plaque.^58^

Based on the overwhelming evidence indicating higher toxicity of the smaller species and the active formation of the larger plaques, we are proposing that the formation of the larger‐insoluble species is an active attempt to restore the dynamic equilibrium of accumulation and clearance of highly toxic small‐diffusible aggregates. This explains the accumulation of large insoluble aggregates with progression of disease, while neurons that fail to form these large aggregates die. However, while the formation of Aβ plaques by microglia have been relatively well understood, the neuronal mechanisms of caspase activation, the mechanisms through which the neurons can survive this, and the succeeding tangle formation needs to be further studied. Lastly, these findings also highlight the importance of single‐molecule and super‐resolution, aggregate‐specific detection techniques^20^, ^21^, ^43^, ^48^, ^59^ as the diffusible aggregates of Aβ and tau are mostly below the diffraction‐limit of light and thus invisible to most immunostaining methods.

If the multi‐stage dynamic equilibria hypothesis is empirically validated, it will have major consequences on our understanding of AD pathophysiology, along with the strategies to diagnose and treat it. We have shown that lecanemab, which is one of the most successful disease modifying treatments, works by predominantly targeting the smaller Aβ aggregates formed during early stages of AD.^19^ Meanwhile, donanemab, which shows no strong binding to soluble species^19^ also produces desirable clinical outcomes through plaque clearance, seemingly to contradicting our hypothesis. While more research is needed to identify the exact working principles of donanemab, it is known that a high concentration of oligomeric Aβ is present around the plaques^58^ and the plaques tagged by donanemab may actually help microglia also clear these smaller aggregates, which may be the main driver of cognitive benefits. Moreover, following biophysical properties of aggregation, removal of plaques that contain a high mass of Aβ would shift the dynamic equilibrium, also leading to the reduction of smaller‐soluble aggregates.

Nonetheless, one major conclusion of our hypothesis is that insoluble plaques and tangles are not the ideal species to monitor the effectiveness of new therapies. For the development of next‐generation therapeutics with even greater success rates than lecanemab and aducanumab, assays evaluating their binding properties to the smaller aggregates and the physiological outcomes of this binding needs to be developed in parallel. While we have developed and utilized advanced single‐molecule detection tools to investigate the binding properties,^19^ Dominic Walsh and colleagues have developed a bioassay using live‐neurons and human AD brain‐derived oligomeric Aβ^60^ to evaluate the potency of monoclonal antibody therapeutics focusing on synaptic deficits as the outcome measure. These types of bioassays indicating the “correct” species to target will be valuable for the development of next‐generation therapeutics. In contrast to Aβ, targeting tau aggregates is more difficult due to their intracellular localization. Our hypothesis predicts that the efficiency of anti‐tau therapeutics can be increased by targeting specific post‐translational modifications. For instance, since the truncation of tau may follow caspase activation and lead to the cessation of this activation, targeting truncated tau may have detrimental effects. Similarly, if the actively formed tangles are protective, targeting them will not be beneficial and instead, the smaller aggregates capable of inducing inflammation should be targeted intra‐ and extra‐cellularly. As such, regardless of further data supporting or disproving the dynamic equilibria hypothesis, instead of targeting Aβ or tau without selectivity for morphologically and bioactivity distinct species, using aggregate type specific assay to identify therapeutics with higher affinity toward disease‐relevant aggregates will lead to the development of next‐generation anti‐AD therapeutics with even higher success rates.

Similar to therapeutic development, novel tools are also needed for the diagnosis of AD based on the detection of soluble aggregate accumulation in early stages, which can then be targeted. Currently available positron emission tomography (PET) imaging and bio‐fluid based biomarker detection techniques predominantly target the larger‐insoluble species, with some newly developed methods targeting the small‐soluble species.^61^, ^62^ Since our work has shown that treatments such as lecanemab would be most beneficial when used at early stages of AD, by targeting the smaller aggregates, diagnostic tools specifically detecting the presence of these species will be highly useful both for drug development and treatment.

Finally, if the multi‐stage dynamic equilibria hypothesis is further validated, a significant number of experimental findings will have to be re‐evaluated. These include seeding experiments in cell cultures using AD brain‐derived tau seeds from the sarkosyl‐insoluble fraction, as the aggregates formed by seeding may be protective rather than toxic. Seeding may actually be a protective mechanism against diffusible‐aggregate toxicity, by inducing tangle formation in functionally connected neurons. This also explains the lack of cell loss in seeding experiments, as we have reported previously.^63^ Similarly, the small‐diffusible aggregates will have to be studied in animal models of AD, using appropriate methods able to detect these aggregates, since the current literature mostly correlates toxicity mechanisms with insoluble plaques and tangles, while the invisible aggregates forming alongside the larger ones may actually be promoting the pathology. Lastly, the implications of the sarkosyl‐insoluble filament structures investigated by CryoEM will have to be re‐evaluated, as these may be the protective species and targeting them, rather than the small‐diffusible aggregates, with immunotherapies could be disadvantageous.^19^, ^47^

## CONFLICT OF INTEREST STATEMENT

Emre Fertan and David Klenerman have no conflicts of interest. Georg Meisl is a consultant for WaveBreak Therapeutics, yet this manuscript has no relationship with Dr. Meisl's role at this company. Author disclosures are available in Supporting Information.

## Supporting information



Supporting Information
